# Protocol of quantitative ultrasound techniques for noninvasive assessing of hepatic steatosis after bariatric surgery

**DOI:** 10.3389/fsurg.2023.1244199

**Published:** 2024-01-04

**Authors:** Bin Chen, Qijie Lu, Bing Hu, Di Sun, Tao Ying

**Affiliations:** ^1^ Department of Ultrasound in Medicine, Shanghai Sixth People's Hospital Affiliated to Shanghai Jiao Tong University School of Medicine, Shanghai, China; ^2^ Shanghai Institute of Ultrasound in Medicine, Shanghai Sixth People's Hospital Affiliated to Shanghai Jiao Tong University School of Medicine, Shanghai, China

**Keywords:** quantitative ultrasound, tissue attenuation imaging, tissue scatter distribution imaging, metabolism-associated fatty liver disease, obesity, Roux-en-Y gastric bypass

## Abstract

**Introduction:**

Roux-en-Y gastric bypass surgery can effectively improve steatosis, necroinflammatory activity, and hepatic fibrosis in individuals diagnosed with morbid obesity or nonalcoholic steatohepatitis (NASH). Common methods such as body mass index (BMI) to evaluate the postoperative effect of clinical bariatric surgery cannot differentiate subcutaneous fats from visceral fats and muscles. Several Quantitative ultrasound (QUS)–based approaches have been developed to quantify hepatic steatosis. QUS techniques (tissue attenuation imaging (TAI), tissue scatter distribution imaging (TSI)) from radio frequency (RF) data analysis as a means for the detection and grading of hepatic steatosis has been posited as an objective and noninvasive approach. The implementation and standardization of QUS techniques (TAI, TSI) in assessing hepatic steatosis quantitatively after bariatric surgery is of high-priority. Our study is aimed to assess hepatic steatosis with QUS techniques (TAI, TSI) in morbidly obese individuals before and after bariatric surgery, and to compare with anthropometric measurements, laboratory assessments and other imaging methods.

**Methods and analysis:**

The present investigation, a self-discipline examination of navigational capacity devoid of visual cues, is designed as a single-site, forward-looking evaluation of efficacy with the imprimatur of the institutional review board. The duration of the study has been provisionally determined to span from 1 January 2023 through 31 December 2025. Our cohort shall encompass one hundred participants, who was scheduled to undergo Roux-en-Y gastric bypass (RYGB) at Shanghai Sixth People's Hospital Affiliated to Shanghai Jiao Tong University School of Medicine. All patients will undergo anthropometric measurements, blood-based biochemical analyses, ultrasonic examination and magnetic resonance imaging proton density fat fraction (MRI-PDFF). The primary endpoint is the analysis of evaluating the efficacy of QUS techniques assessing hepatic steatosis compared to other methods before and after bariatric surgery.

**Results:**

Prior to the fomal study, we recruited 21 obese Chinese participants who received ultrasonic examination (TAI, TSI) and MRI-PDFF. AC-TAI showed moderate correlations with MRI-PDFF (adjusted *r* = 0.632; *P* < 0.05). For MRI-PDFF ≥10%, SC-TSI showed moderate correlations with MRI-PDFF (adjusted *r* = 0.677; *P* < 0.05).

**Conclusion:**

Our pre-experiment results signified that using QUS techniques for postoperative evaluation of bariatric surgery is promising. QUS techniques will be signed a widespread availability, real-time functionality, and low-cost approach for assessing hepatic steatosis before and after bariatric surgery in obese individuals, thus is capable for subsequent scale-up liver fat quantification.

**Ethics and dissemination:**

The present research endeavor has been bestowed with the imprimatur of the Ethics Committee of the Hospital, as indicated by its Approval Number: 2023-KY-015. In due course, upon completion of the study, we intend to disseminate our findings by publishing them in a suitable academic journal, thereby facilitating their widespread utilization.

**Registration:**

The trial is duly registered with the Chinese Clinical Trial Registry, and with a unique Trial Registration Number, ChiCTR2300069892, approved on March 28, 2023.

## Introduction

Obesity has emerged as a chronic health condition that has garnered significant attention from the international community. The World Health Organization's latest report indicates a staggering prevalence of over 1.9 billion overweight adults globally, with over 650 million individuals classified as obese. The gravity of this epidemic demands immediate and sustained action to mitigate the social, economic, and health consequences associated with obesity (1). Obesity is associated with numerous comorbidities, such as metabolism-associated fatty liver disease (MAFLD), cardiovascular diseases, type 2 diabetes, hypertension, and dyslipidemia. Hepatic steatosis is commonly associated with obesity, and its prevalence can be as high as 80% in extremely obese individuals (2). In a study with 1,000 morbidly obese patients, more than 95% had MAFLD (3). The contribution of obesity and chronic low-grade systemic inflammation to the progression from hepatic steatosis to fibrosis is a matter of considerable significance. These factors are known to exert significant influence on the development of liver disease, thus warranting further scrutiny in this context (4). The recalcitrance of obesity portends a surge in the incidence of MAFLD, and as this cohort advances in age, a considerable proportion will encounter hepatic cirrhosis and end-stage liver maladies.

Severe obesity presents as a chronic condition that is resistant to treatment. While it is commonly believed that a combination of dietary adjustments and heightened physical exertion may yield considerable long-term advantages, such efforts prove challenging to uphold. Regrettably, over 95% of patients who attempt conservative interventions fail to attain desired outcomes. Bariatric surgery may be more effective than medical and lifestyle interventions for the most severe form of obesity to have major and sustainable weight loss, with RYGB being the most popular surgical procedure (5). Patients undergoing this surgery not only lose a substantial amount of weight, but also are clearly improved in clusters of the metabolic syndrome.

The widespread employment of BMI as a metric for assessing the postoperative efficacy of clinical bariatric surgery is an established practice. However, this measure fails to discriminate between subcutaneous adipose tissue and visceral fat as well as muscle mass (6). The changes of laboratory values (like ALT, AST, HDL-c and LDL-c) after surgery are also the point of assessment of surgical effect (7), but lack of standardization. While liver biopsy remains the gold standard for diagnosing and grading hepatic steatosis, its invasiveness, lack of reproducibility, and potential complications preclude its use in ongoing monitoring efforts. The accurate grading of hepatic steatosis is crucial for the appropriate management of patients. While MRI-PDFF estimation offers a noninvasive alternative to liver biopsy, its high cost and limited availability constrain its global implementation (8–10). As an alternate noninvasive option, ultrasound (US) holds promise due to its widespread access and cost-effectiveness. B-mode US imaging based on the amplitude of the envelope of beamformed radiofrequency signals is routinely employed in clinics but suffers from operator-dependency, subjectivity, and low sensitivity to mild steatosis (11). While conventional US shows sensitivity and specificity ranges of 60%–94% and 66%–95%, respectively, it does not offer an adequate diagnostic tool for quantifying hepatic steatosis. Nonetheless, recent efforts have been made to develop QUS, which has the potential to enhance diagnostic accuracy and enable liver fat quantification (12, 13). QUS represents a promising application of this modality that may improve the diagnosis and management of hepatic steatosis.

**Figure 1:**
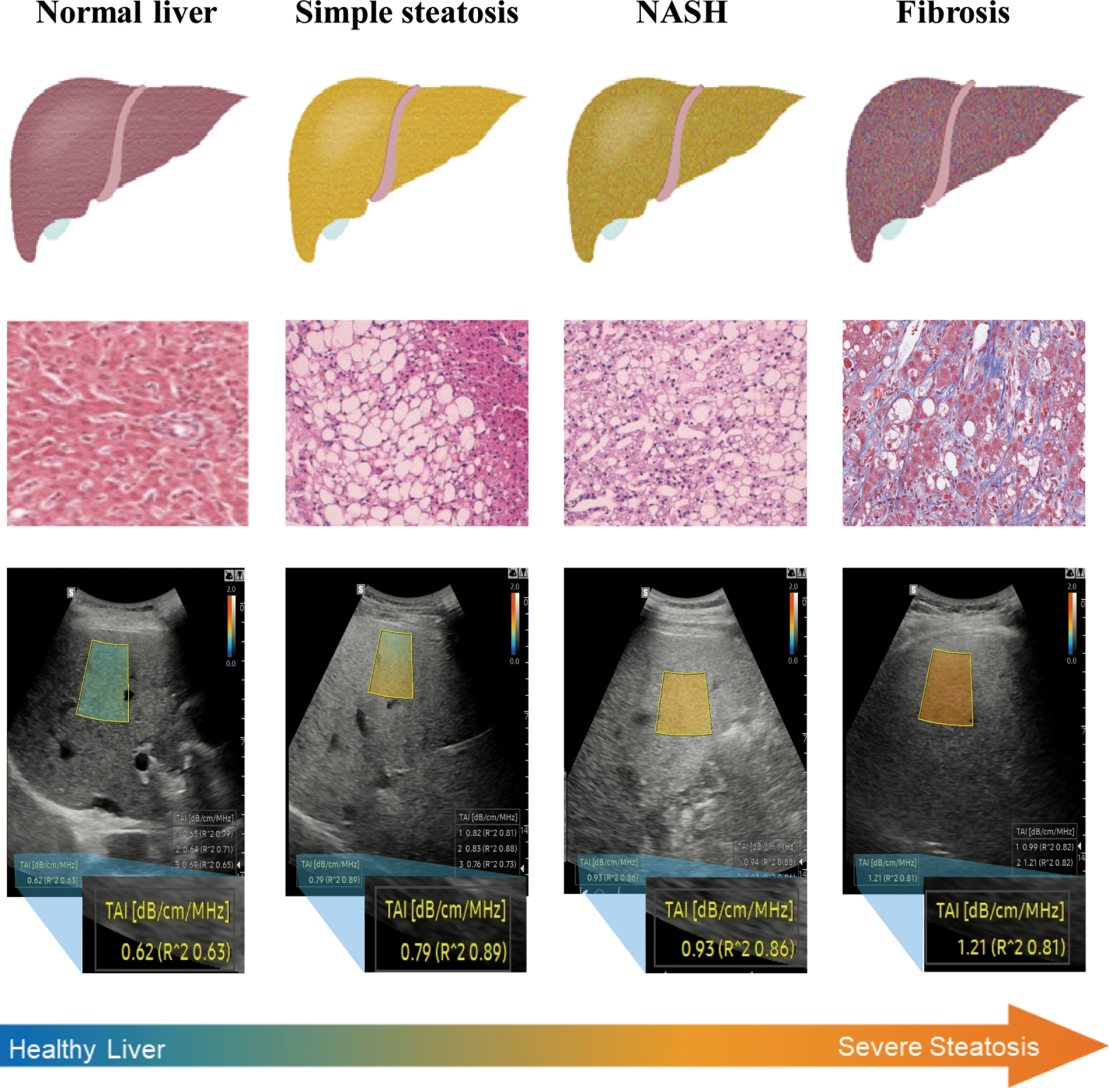
NAFLD is a progressive disease, ranging from simple steatosis to NASH and cirrhosis. QUS techniques show good diagnostic performance for hepatic steatosis detection in obese individuals. QUS parameters (SC-TSI) show a significant correlation with hepatic steatosis (19, 20).

In recent years, several QUS techniques have emerged as promising non-invasive approaches for assessing hepatic steatosis. These techniques employ non-image-based parameters such as the speed of sound, ultrasound attenuation, backscatter coefficient, and shear-wave dispersion to evaluate liver fat content, and have demonstrated a strong correlation with hepatic steatosis in previous studies (11, 14, 15). Despite their potential benefits, however, these QUS techniques also have certain limitations. For example, the hepatorenal index, which measures the ratio of liver to kidney echointensities on B-mode ultrasound, can quantitatively assess hepatic steatosis but may not be suitable for patients with renal disease or those with uneven distribution of steatosis (16). Controlled attenuation parameter(CAP) is only available on FibroScan, and this technique does not provide B-mode image during examing. To overcome these issues, researchers have proposed using quantitative US parameters derived from RF data analysis. Two such parameters include the attenuation coefficient (AC) based on TAI (AC-TAI) and scatter-distribution coefficient (SC) based on TSI (SC-TSI). AC-TAI indicates the slope of the US center frequency downshift with a depth that can be used to estimate acoustic attenuation (17). SC-TSI, on the other hand, reflects the local concentration and arrangement of US scatterers by indicating the average Nakagami parameters of the region of interest (ROI) (18). Unlike B-mode images, raw RF data contain frequency-dependent information of the US signal and offer additional diagnostic value. These data can be obtained by activating a specific acquisition mode. By using these parameters, researchers have demonstrated improved accuracy in diagnosing steatosis grades compared to qualitative visual scores on B-mode ultrasound (Figure 1).

The protocol is aimed to provide a method to assess hepatic steatosis with QUS techniques (TAI, TSI) in morbidly obese individuals before and after bariatric surgery, and to compare with anthropometric measurements, laboratory assessments and other imaging methods. Thereby, the application value of QUS techniques (TAI, TSI) in quantitatively assessing hepatic steatosis after bariatric surgery will be clarified.

## Methods and analysis

### Design

In order to enhance the lucidity of our study's design, we have formulated a succinct PICOT framework consisting of the following components.

### P (patient, population or problem)

Our investigation shall encompass a cohort of 100 patients who are slated to undergo RYGB at Shanghai Sixth People's Hospital Affiliated to Shanghai Jiao Tong University School of Medicine.

### I (intervention)

All patients will receive anthropometric measurements (BMI, waist circumference, hip circumference), blood-based biochemical analyses (alanine aminotransferase ALT, aspertate aminotransferase AST, γ-glutamine transferase γ-GTP, total cholesterol Tc, triglyceride, high-density lipoprotein cholesterol HDL-c, low-density lipoprotein cholesterol LDL-c), ultrasonic examination (B-mode US, CAP, TAI, TSI) and magnetic resonance imaging proton density fat fraction (MRI-PDFF) before and 1, 3, 6, 12 months after bariatric surgery.

### C (comparison or control)

Our research constitutes a prospective superiority trial, wherein self-control and blind map-reading served as key components. The study was conducted within a single-center and aimed to assess the efficacy of QUS techniques compared to other imaging methods, anthropometric measurements, and laboratory assessments which were designated as control groups.

### O (outcome or objective)

**Figure 2:**
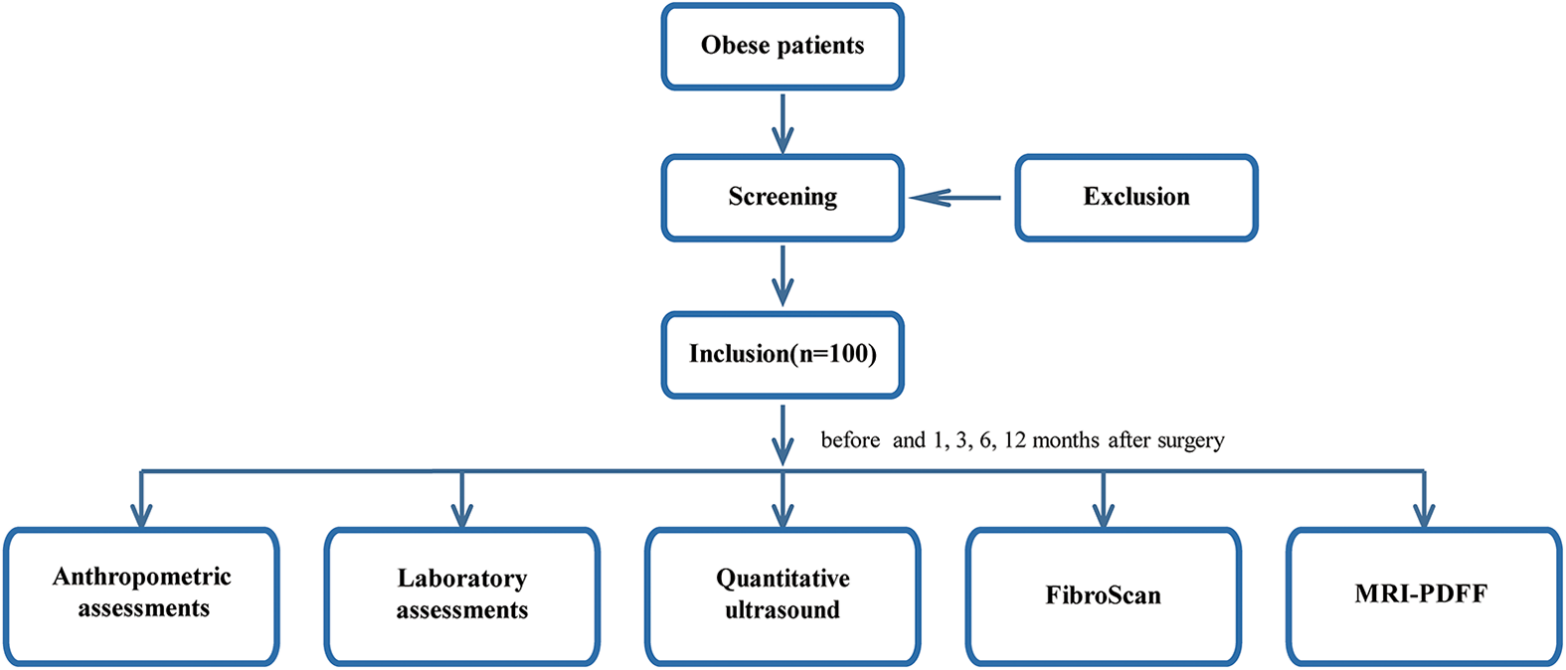
The study flow diagram.

The focal point of the study resides in evaluating the efficacy of QUS techniques assessing hepatic steatosis, utilizing MRI-PDFF as the gold standard, compared to other methods before and after bariatric surgery. The secondary endpoint focuses on evaluation of hepatic steatosis and its changes in obese individuals before and after bariatric surgery using QUS techniques (TAI, TSI) over a period of 1, 3, 6, and 12 months. The tertiary endpoint aims to observe the changes of indicators (anthropometry, laboratory assessment, imaging examination) before and after bariatric surgery, and compare the correlation between QUS and traditional evaluation indices. The study flow diagram is presented in Figure 2.

### T (time frame)

Upon obtaining consent from the institutional review committee, it has been determined that the designated period of investigation will commence on the first day of January in the year 2023 and conclude on the last day of December in the year 2025.

## Study participants

### Inclusion criteria

The parameters for eligibility include three criteria all of which must be satisfied:

(1) age between 18 and 85 years old;
(2) BMI ≥ 27.5 kg/m^2^ and scheduled to undergo RYGB;
(3) willingness and ability to participate.

### Exclusion criteria

(1) age < 18 or >85 years old;
(2) the use of hepatotoxic or steatogenic medication;
(3) excessive alcohol consumption (14 and 7 drinks per week for men and women, respectively);
(4) previous liver surgery;
(5) MRI contraindications (due to claustrophobia, metal implants, or body circumference beyond the aperture of the MRI scanner);
(6) major systemic diseases.

### Schedule of the study

Upon securing informed consent, the cohort shall meticulously evaluate the criteria for inclusion and exclusion as well as background information pertaining to each participant. It is noteworthy that a total of 104 cases were scrutinized in this study, consisting of 100 formal cases and four training cases, which were consecutively incorporated into the analysis. All participants will receive anthropometric measurements (BMI, waist circumference, hip circumference), blood-based biochemical analyses (ALT, AST, γ-GTP, Tc, Triglyceride, HDL-c and LDL-c), ultrasonic examination (B-mode US, CAP, TAI, TSI) and MRI-PDFF.

### Case screening period

In accordance with the criteria of inclusion and exclusion, subjects are consecutively enrolled and essential data is gathered subsequent to securing informed consent.

### After inclusion

This study includes anthropometric measurements, blood-based biochemical analyses, ultrasonic examination and MRI-PDFF. All parts are completed independently in a blind state. There is no specific requirement on the sequence of the examinations. All examinations shall be conducted on the same day. All participants were educated after inclusion, and they were told to maintain a reasonable and stable lifestyle as much as possible after surgery, so as to avoid experimental error from unreasonable lifestyle (such as fasting, overeating).

### Anthropometric and laboratory assessments

Anthropometric and laboratory evaluations were conducted as part of the study protocol. Trained physicians performed subject interviews to elicit medical history, medication usage, and alcohol consumption habits. Afterward, anthropometric measurements were taken while subjects were attired in lightweight garments and shoeless. These measurements included height, weight, waist circumference (WC), and hip circumference (HC). Body Mass Index (BMI) was then computed using the individual's weight divided by the square of their height (kg/m^2^). Laboratory assessments were evaluated by obtaining serum samples after an overnight fast and were as follows: ALT, AST, γ-GTP, Tc, Triglyceride, HDL-c and LDL-c.

### B-mode US

For each participant, B-mode US examination was performed using a US system (RS 85, Samsung Medison, Co. Ltd.) with a convex probe (CA1-7A). All participants were requested to fast for at least 8 h before the US examinations. Hepatic steatosis will be graded subjectively by the operator as no steatosis, mild steatosis, moderate steatosis, severe steatosis. They were be graded based on the following findings: (1) higher echogenicity of the liver, (2) impaired visualization of the intrahepatic vessels, and (3) impaired visualization of the diaphragm and posterior right hepatic lobe due to ultrasound beam attenuation (21).

### QUS techniques

The QUS technique was utilized to evaluate each participant in this study. A US system with a convex probe was used to conduct the QUS analysis (RS 85, Samsung Medison, Co. Ltd.). Two QUS parameters, namely AC-TAI and SC-TSI, were calculated from the RF data using an inhouse program developed in MATLAB R2015a (MathWorks, Inc.). The color-coded maps of both AC-TAI and SC-TSI were produced by analyzing the RF data of the corresponding B-mode images.

To determine the TAI and TSI maps of the liver parenchyma, one researcher carefully placed ROIs (approximately 2 cm in inner arc length and 4 cm in height) on the B-mode images while avoiding large vessels, focal lesions, and reverberation artifacts under the liver capsule. In cases where blood vessels were unavoidable, the areas containing large vessels were excluded from the calculation of AC-TAI and SC-TSI, which resulted in vacant regions on the TAI and TSI maps. The MRI-PDFF results were unknown to the researchers when measuring the QUS parameters. The five measurements for each examination were averaged for each QUS parameter.

### CAP

The assessment of hepatic steatosis in the present study was carried out using FibroScan® (Echosens, Paris, France), with X- or XL-probes employed to capture the CAP in dB/m. The examination process involved instructing all participants to fast for a minimum of 6 h before assuming a supine position with their right arm in maximal abduction. The technique entailed scanning the right liver lobe through an intercostal space and aiming to obtain ten successful acquisitions from each subject. Trained operators executed the transient elastography while remaining masked to data and participant diagnoses. The final CAP score (dB/m) was derived from the median of individual measurements, with the quartile spacing of CAP not exceeding 40 dB/m.

### MRI

All participants underwent liver MRI using chemical shift encoding. To measure proton density fat fraction (PDFF), the researchers employed complex-based chemical shift-encoded water-fat reconstruction techniques with six two-dimensional gradient-recalled-echo images, an imaging matrix of 256 × 192, and a 3 mm slice thickness. A low flip angle (4°) was used to minimize the T1 bias between fat and water. The PDFF maps were reconstructed automatically using the vendor's algorithm with T2* correction calculated from signal decay and a multi-peak fat model. For each patient, one experienced radiologist, blinded to the other test and examination results, manually demarcated the whole liver boundary of the PDFF map. The section chosen to demarcate need avoid large vessels and focal lesions as far as possible. The average of ROIs was used as the reference standard for hepatic fat content.

### Image assessment

The images obtained during the study have been saved as image files. The evaluators will be required to assess the level of hepatic steatosis without any clinical data. After deliberation and achieving a consensus, the evaluators will complete the case report forms (CRFs). Subsequently, the study coordinators shall gather all images and CRFs for specialized storage within the database.

### Primary endpoint

In this study, the primary endpoint seeks to evaluate the efficacy of QUS techniques assessing hepatic steatosis, utilizing MRI-PDFF as the gold standard, compared to anthropometric measurements, laboratory assessments and other imaging methods before and after bariatric surgery. Success in this endeavor would be measured by the ability of QUS techniques to better evaluate the degree of hepatic steatosis as compared to other methods, thereby providing diagnostic improvement beyond current practice. Such results would signify the feasibility of using QUS techniques for postoperative evaluation of bariatric surgery.

### Secondary endpoint

The secondary endpoint focuses on evaluation of hepatic steatosis and its changes in obese individuals before and after bariatric surgery using QUS techniques (TAI, TSI) over a period of 1, 3, 6, and 12 months. Interpretation of the data collected will depend on whether significant differences are observed in QUS parameters pre- and post-surgery. The confirmation of such differences would furtherly signify the feasibility of using QUS techniques for postoperative evaluation of bariatric surgery. In addition, the trend in the changes of hepatic steatosis would evaluate the influence of bariatric surgery on obese individuals.

### Tertiary endpoint

Finally, the tertiary endpoint aims to observe the changes of indicators (anthropometry, laboratory assessment, imaging examination) before and after bariatric surgery, and compare the correlation between QUS and traditional evaluation indices. It may indicate whether there are differences in these indicators before and 1, 3, 6 and 12 months after bariatric surgery, and these differences will assess the influence of bariatric surgery on these indicators.

## Stopping rules

(1) Patient's voluntary withdrawal;
(2) The subject's request to withdraw their informed consent;
(3) Medical necessity for discontinuation of the study by the researchers, etc.

### Sample size

To ensure accurate evaluation of QUS techniques for hepatic steatosis grading, it is imperative to calculate the required sample size. Assuming an 80% MAFLD prevalence in the target population and 80% sensitivity and 90% specificity in QUS techniques, we calculated the minimum number of subjects needed to be 75 using a paired non-parametric test in the non-parametric module of PASS. Considering factors like data loss, cases “lost to follow-up”, our study will involve 100 patients scheduled to undergo RYGB.

### Data management

For maintaining personal information confidentiality, enrolled patients will be assigned a unique identification number that will replace their real names throughout the clinical project. A trained researcher will document and submit data accuracy and completeness via CRFs to an expert committee for review. The collected data will be entered into a specialized database through an electronic CRF for secure storage. When all participants complete the study, the dataset will be analyzed accordingly.

### Data analysis

The statistical analysis for this study will be performed using the SAS 9.2 software package (SAS Analytics, Marlow, UK) and R (v4.3.1; R Core Team), which is widely available for commercial use. The significance level has been predetermined to be 0.05 for all two-sided tests. The mean and standard deviation will be used to express normally distributed quantitative data.

To compare quantitative data, a paired t-test will be employed. Meanwhile, categorical variables will be analyzed using the chi-squared test. Pearson correlation coefficients will be utilized to evaluate the relationship between QUS parameters and other factors under consideration. Univariable linear regressions of QUS and other methods on MRI-PDFF were conducted to calculate the adjusted linear correlation coefficient (r). Then receiver operating characteristic (ROC) curves of QUS and other methods for the detection of MRI-PDFF ≥5% and ≥10% were plotted. The best cut-off values were defined as the cut-off values corresponding to the maximum Youden indexes.

## Results

**Figure 3:**
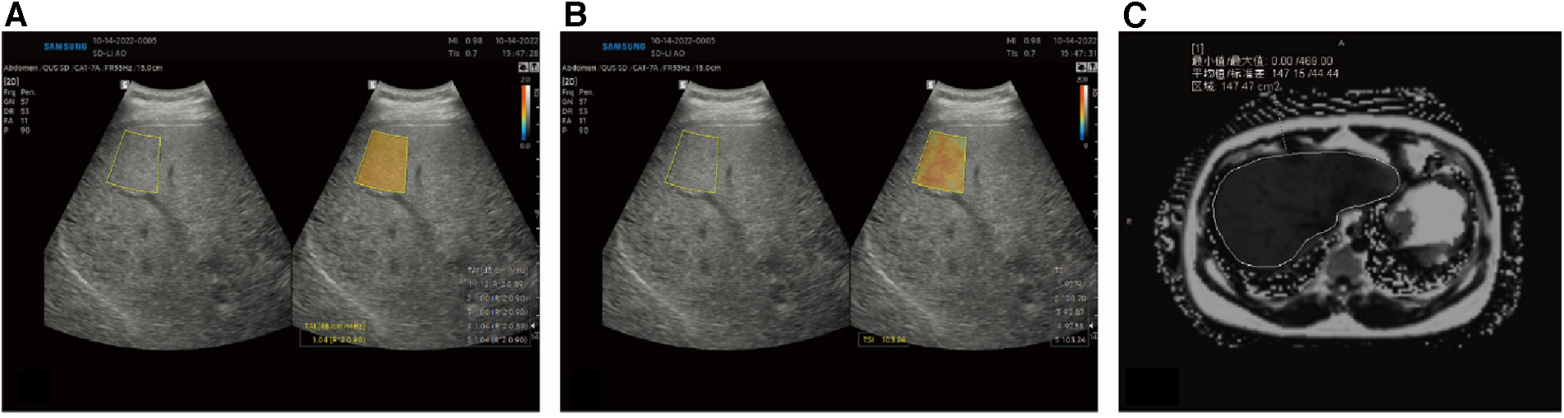
Quantitative analysis of liver fat in an obese Chinese male. Based on B-mode ultrasound image, the color-coded maps of TAI map (**A**) reflecting center frequency and TSI map (**B**) reflecting Nakagamia parameters are generated. (**C**) MRI-PDFF values were 14.72%.

Prior to the fomal study, we recruited 21 Chinese participants who satisfied: (1) age between 18 and 85 years old; (2) with suspected NAFLD, and (3) agreed to receive QUS, and MRI examination. They received ultrasonic examination (TAI, TSI, CAP) and MRI-PDFF (Figure 3).

The average BMI of all participants was 26.43, which meets the diagnostic criteria for overweight. More than 50% of patients (15/21, 71.43%) presented with symptom of right upper abdominal distension. For the value of MRI-PDFF, the average was 13.86%. MRI results from eight patients were below 10%. For MRI-PDFF ≥10%, a total of 13 patients were in this category (13, 62.9%).

**Figure 4:**
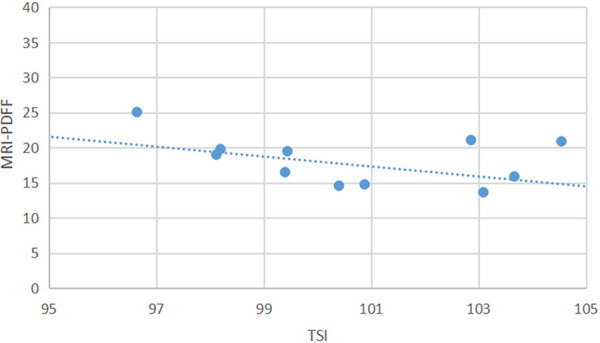
Scatterplot of SC-TSI and MRI-PDFF.

All the data were analyzed in R (v4.3.1; R Core Team). A two-sided *P* < 0.05 was considered statistically significant. AC-TAI showed moderate correlations with MRI-PDFF (adjusted *r* = 0.632; *P* < 0.05). Linear correlation between SC-TSI and MRI-PDFF was not significant (*P* = 0.982) (Table 1). For MRI-PDFF ≥10%, SC-TSI showed moderate correlations with MRI-PDFF (adjusted *r* = 0.677; *P* < 0.05) (Figure 4). In a way, the results signified the feasibility of using QUS techniques for postoperative evaluation of bariatric surgery.

**Table 1:** Linear regression between AC-TAI/SC-TSI and other parameters.

Parameters	AC-TAI		SC-TSI	
	Adjusted R	*P*-value	Adjusted R	*P*-value
CAP	0.578	<0.05	0.343	0.070
MRI-PDFF	0.632	<0.05	0.229	0.982

## Discussion

The rising prevalence of obesity has emerged as a significant public health challenge across the world, affecting both developed and developing nations. Among the numerous adverse consequences of obesity, an important correlation has been established between its degree and the incidence and severity of MAFLD. In fact, obesity has been found to increase the likelihood of developing MAFLD by 4.6 times (22). The natural course of MAFLD indicates that fibrosis manifests in around 32–37% of patients within 3–6 years, and approximately 12% of patients tend to progress to cirrhosis over 8–10 years (23).

Therapeutic interventions such as bariatric surgery have demonstrated potential in ameliorating steatosis, hepatic fibrosis, and necroinflammatory activity, especially for those with morbid obesity and NASH. Weiner et al. (24) conducted a study to evaluate the impact of bariatric surgery on the histological development of NASH. They discovered, through liver biopsy results, that 82.8% of patients exhibited complete regression of MAFLD. Dixon et al. (25) investigated the outcomes of weight loss following Roux-en-Y gastric bypass (RYGB) on liver histology and plasma aminotransferase levels (AST, ALT, and GGT) in 60 severely obese individuals. Results indicated that the mean weight loss was 31.5 ± 18 kg, and there was notable improvement in AST, ALT, GGT, lobular steatosis, inflammation, and fibrosis. Furuya et al. (26) enrolled a cohort of MAFLD patients undergoing RNYGB, and procured a wedge liver biopsy during the operation. The initial evaluation revealed that 67% of the patients had NASH, while 33% had steatosis, and cirrhosis was present in 5.5% of those with NASH. Following an average excess weight loss of 60%, steatosis and fibrosis disappeared in 84% and 75% of the patients respectively.

Continuous surveillance of hepatic steatosis following bariatric surgery is crucial for effective disease management and prompt therapeutic intervention, ultimately leading to improved health outcomes and reduced healthcare costs (10). Recently, several QUS approaches have emerged for the quantification of hepatic steatosis. The advantages of a US-based hepatic fat quantification system are numerous, including widespread availability, real-time functionality, and low cost. Recent studies have demonstrated that certain quantitative parameters derived from RF data analysis, which reflect the backscatter or attenuation of US beams, exhibit strong correlation with hepatic steatosis grades (27–29). Jeon et al. (30) investigated the diagnostic potential of QUS parameters in assessing hepatic steatosis among MAFLD patients, using MRI-PDFF as the reference standard. They found that both AC-TAI and SC-TSI were significantly correlated with MRI-PDFF (*r* = 0.659 and 0.727, respectively). For detecting hepatic fat contents ≥ 5%, the areas under the receiver operating characteristic (ROC) curves of AC-TAI and SC-TSI were 0.861 and 0.964, respectively. Bae et al. (31) on the other hand, evaluated the diagnostic performance of the normalized local variance ultrasound technique in detecting fatty liver, using histopathology as a reference standard. The areas under the ROC curve for detecting fatty livers of varying degrees were 0.911 for ≥S1, 0.974 for ≥S2, and 0.954 for ≥S3. This study aims to provide evidence supporting the feasibility of utilizing QUS techniques for liver fat quantification. Furthermore, we aim to prospectively evaluate the application value of QUS techniques in quantitatively assessing hepatic steatosis after bariatric surgery (32, 33).

Pereira et al. (34) conducted a comparison of noninvasive biochemical methods and FibroScan with histopathologic analysis to evaluate hepatic fibrosis in patients who underwent bariatric surgery one year prior. The results indicate that noninvasive liver fibrosis evaluation correlates highly with liver biopsy findings after one year of bariatric surgery. While QUS techniques (TAI, TSI) are more advanced ultrasound technologies, they can theoretically assess the degree of hepatic steatosis accurately after bariatric surgery. In our pre-experiment study, we preliminarily verified the feasibility of the QUS techniques in Chinese participants who suspected NAFLD. The results of the pre-experiment signified the feasibility of using QUS techniques for postoperative evaluation of bariatric surgery, making our study design reasonable.

Our study inevitably has some limitations. Specifically, we utilized MRI-PDFF rather than biopsy as the gold standard to correlate QUS parameters. Although previous research suggests that MRI-PDFF exhibits relatively high sensitivity and specificity in detecting fatty liver disease, there remains potential for incorrect diagnoses. In addition, although we conducted health education for participants before surgery, it was difficult to avoid that confounding factors (like life-style) may influence on results. Furthermore, confounding factors such as increased subcutaneous fat in individuals with higher BMI may influence QUS parameter results. With a larger patient population, we will summarize the imaging experience in hepatic steatosis evaluation by QUS techniques in obese people. We will also analyze the influence of scanning methods, imaging location and other influencing factors on the results, and explore how to improve the results.

## Conclusion

In conclusion, this article has described methods for using QUS techniques (TAI, TSI) to assess hepatic steatosis and its changes in obese individuals before and after bariatric surgery. In addition, we compare QUS techniques to anthropometric measurements, laboratory assessments and other imaging methods. By this study, QUS techniques will be signed a widespread availability, real-time functionality, and low cost approach for assessing hepatic steatosis before and after bariatric surgery in obese individuals. Our pre-experiment results signified that using QUS techniques for postoperative evaluation of bariatric surgery is promising. Our study is the first one in China to investigate the application value of QUS techniques (TAI, TSI) in quantitatively assessing hepatic steatosis after bariatric surgery. Our study will provide strong evidence regarding whether obese people can truly benefit from QUS techniques in liver fat quantification.

## Data Availability

The original contributions presented in the study are included in the article/Supplementary Material, further inquiries can be directed to the corresponding authors.
